# Duodeno-cholecystostomy

**Published:** 1889-10

**Authors:** 


### DUODENO-CHOLECYSTOSTOMY.

A letter from Dr. F Ferrier, of Paris, to Dr. J. McFadden Gaston, of this city, contains the following statement:

"I have made a fistula between the gall-bladder and duodenum on a woman, and I hope to have a very good result. The operation has been made in one time, and by a proceeding corresponding to your indications on the dog. I shall send you the number of the Revue de Chirurgie, in which I will publish this observation."

This successful issue of Gaston's operation for the return of the bile to the alimentary canal, in occlusion of the common bile duct, entitles him to recognition as the author of this great improvement in surgery. It will afford us pleasure to transfer to our pages the report of Dr. Ferrier, when it appears in the French Review.

